# Sustainable repurposing of disposable SMS polypropylene gowns in dental education: a feasibility study on material functionality and user perception

**DOI:** 10.3389/fdmed.2026.1778387

**Published:** 2026-07-20

**Authors:** Franz Tito Coronel-Zubiate, Jeanile Zuta-Rojas, Oscar Pizarro-Salazar, Susan Yesabel Bustamante-Visalot, Erikson Alexander Jimenez-Torres, Heber Isac Arbildo-Vega

**Affiliations:** 1 Faculty of Health Sciences, Stomatology School, Universidad Nacional Toribio Rodriguez de Mendoza de Amazonas, Chachapoyas, Peru; 2 Faculty of Dentistry, Dentistry School, Universidad San Martin de Porres, Chiclayo, Peru; 3 Faculty of Human Medicine, Human Medicine School, Universidad San Martín de Porres, Chiclayo, Peru; 4 Graduate School, Universidad Nacional Toribio Rodriguez de Mendoza de Amazonas, Chachapoyas, Peru

**Keywords:** circular economy, dental education, environmental sustainability, green dentistry, healthcare waste management, material repurposed, non-critical clinical barriers, SMS polypropylene

## Abstract

**Background:**

The widespread use of disposable polypropylene-based personal protective equipment (PPE) in dentistry contributes significantly to healthcare waste. Sustainable approaches that promote responsible material use within educational clinical settings are increasingly needed to mitigate the environmental footprint of dental training.

**Objective:**

To evaluate the feasibility of a controlled, sustainability-oriented repurposing strategy for disposable SMS polypropylene surgical gowns in a university dental clinic, focusing on functional performance, user perception, and educational implications.

**Materials and methods:**

A mixed-methods, quasi-experimental applied study was conducted between August and November 2025. Used SMS gowns underwent standardized inspection, cleaning, disinfection (0.1% sodium hypochlorite), terminal dry heat treatment (70–75 °C), and subsequent transformation into repurposed dental chair covers for use as non-critical barriers during non–aerosol-generating procedures. Functional performance was assessed via structured checklists. User perception and instrument reliability (*α* = 0.82) were evaluated through validated 5-point Likert-scale questionnaires administered to dental students (*n* = 148) and professionals (*n* = 25). This study does not aim to validate microbiological sterility or clinical repurposing of personal protective equipment, but rather to assess sustainability-oriented repurposing into non-critical barriers within an educational dental setting.

**Results:**

Of 286 gowns collected, 205 (71.7%) met eligibility criteria and were successfully repurposed. The transformed materials demonstrated adequate stability, resistance to displacement, and tolerance to routine surface disinfection. High acceptance was reported across both groups (Global Index: 3.89/5), with significantly stronger institutional adoption support among professionals (80%) compared to students (66.2%). Preliminary environmental estimation further indicated that the intervention diverted approximately 12.9 kg of SMS polypropylene from disposal, corresponding to an estimated reduction of 20.5 kg CO₂-equivalent emissions.

**Conclusions:**

Controlled repurposing of disposable SMS gowns into non-critical barriers is feasible and represents a practical strategy to reduce clinical waste while fostering sustainability competencies in dental education. By integrating functional assessment, stakeholder acceptance, and preliminary environmental impact estimation within a real-world academic workflow, this study provides a replicable systems-integration model that supports the implementation of circular economy principles in dental education.

## 1 Introduction

The increasing generation of healthcare waste has become a major global environmental concern, driven largely by the widespread use of disposable materials made from non-biodegradable polymers. Among these materials, polypropylene-based personal protective equipment (PPE), including disposable surgical gowns, contributes significantly to the accumulation of clinical waste in healthcare facilities. The environmental impact of these materials is particularly relevant due to their high consumption and limited recovery options after use (1, 2).

In dental settings, strict infection control protocols require the routine use of disposable protective barriers to ensure patient and operator safety. As a result, university dental clinics and outpatient care facilities generate considerable volumes of PPE-related waste on a daily basis (3). The reliance on single-use surgical gowns, while effective in maintaining biosafety, has intensified the environmental footprint of dental clinical practice, especially in academic environments characterized by continuous patient care and student training activities (4, 5).

Following the COVID-19 pandemic, the use of disposable PPE increased substantially, reinforcing biosafety measures but also exacerbating waste generation in healthcare facilities and dental schools (6–9). While disposable surgical gowns provide effective protection during clinical procedures, their short lifespan and immediate disposal after a single use raise concerns regarding sustainability, resource consumption, and waste management (10, 11). These concerns are particularly relevant in university dental clinics, where high patient turnover and continuous training activities result in considerable volumes of discarded materials.

In response to these challenges, sustainability-oriented strategies such as repurposing, material optimization, and circular economy principles have gained increasing attention in the healthcare sector. Controlled repurposing approaches (12), when conducted under standardized inspection and handling protocols, have been proposed as potential strategies to reduce waste generation and improve resource efficiency. Rather than focusing exclusively on microbiological validation of critical items, this sustainability-centered initiative emphasizes responsible material use for non-critical clinical barriers, environmental stewardship, and the educational value of integrating sustainable practices into clinical training (13, 14).

Universities play a crucial role in fostering environmental awareness and responsible professional behavior among students in health sciences. Incorporating sustainability initiatives into clinical training not only contributes to waste reduction but also encourages critical reflection on material use, biosafety practices, and environmental impact. In dental education, experiential learning projects focused on repurposing and sustainability may enhance students' understanding of environmental stewardship while maintaining adherence to established biosafety and infection control frameworks (15–17).

Despite growing interest in sustainable healthcare practices, there is limited evidence addressing the feasibility and educational impact of repurposing disposable surgical gowns in dental settings, particularly from an applied and environmentally focused perspective (18). Most existing studies prioritize laboratory-based microbiological assessments or life cycle comparisons of disposable and reusable systems, leaving a gap in the literature regarding real-world implementation within academic clinical environments. Furthermore, few studies have simultaneously integrated functional evaluation, stakeholder perception, and quantitative environmental assessment into a single operational framework for dental education. This study addresses these gaps by evaluating the functional repurposing of PPE into non-critical barriers while integrating environmental sustainability, resource optimization, and educational implementation within routine clinical training. In doing so, it extends recent calls to embed circular economy principles into healthcare education through practical, institution-based sustainability interventions.

Therefore, the aim of this study was to evaluate the feasibility of a controlled, sustainability-oriented repurposing strategy for disposable SMS polypropylene gowns in a university dental clinic. Beyond simple material repurposing, the study integrates functional performance assessment, user perception analysis, and a preliminary environmental impact estimation within an academic clinical workflow. By operationalizing circular principles at the institutional level, rather than focusing solely on laboratory-based validation, this work proposes a replicable systems-integration model for sustainable dental education and clinical practice. This study does not aim to establish microbiological sterility or support clinical reuse of PPE; rather, it addresses sustainability-oriented repurposing into non-critical environmental barriers within dental education settings.

## 2 Materials and methods

### 2.1 Study design and ethical approval

This applied study adopted a mixed-methods, quasi-experimental design aimed at evaluating the feasibility of a sustainability-focused repurposing strategy for disposable SMS polypropylene surgical gowns in a university dental setting. The protocol was approved by the Institutional Research Ethics Committee of the National University Toribio Rodríguez de Mendoza de Amazonas (approval code: CIEI-00248) and conducted between August 2025 and November 2025 as part of an institutional sustainability initiative.

### 2.2 Study setting and participants

The intervention was carried out at the university dental clinic and an adjacent textile-preparation area. These facilities allowed adequate separation between clinical use, material inspection, cleaning/disinfection, and transformation processes, ensuring controlled handling conditions throughout all stages of the intervention.

Two groups were included: undergraduate dental students (preclinical and clinical levels) and dental professionals (faculty members and clinicians). A non-probabilistic convenience sampling approach was employed. Eligibility required direct exposure to the intervention, either through participation in the transformation process or use of the reprocessed materials during non-critical environmental barrier applications within clinical activities. Participation was voluntary and anonymous. In total, 148 students and 25 professionals completed the perception surveys.

### 2.3 Materials

Disposable SMS polypropylene gowns were collected after a single clinical use. Only gowns strictly free of visible biological contamination, structural damage, discoloration, or odor were included to ensure the safety of the transformation process and prevent inappropriate reuse. Materials failing these criteria were discarded following institutional biosafety and waste management policies.

Disinfection supplies included neutral detergent, freshly prepared 0.1% sodium hypochlorite solution, sterile water, and controlled air-drying facilities. Textile transformation was performed using standard sewing equipment, reinforced polyester thread, elastic bands or adjustable ties, and standardized templates adapted to dental chair dimensions.

### 2.4 Repurposing and transformation protocol

**Figure 1:**
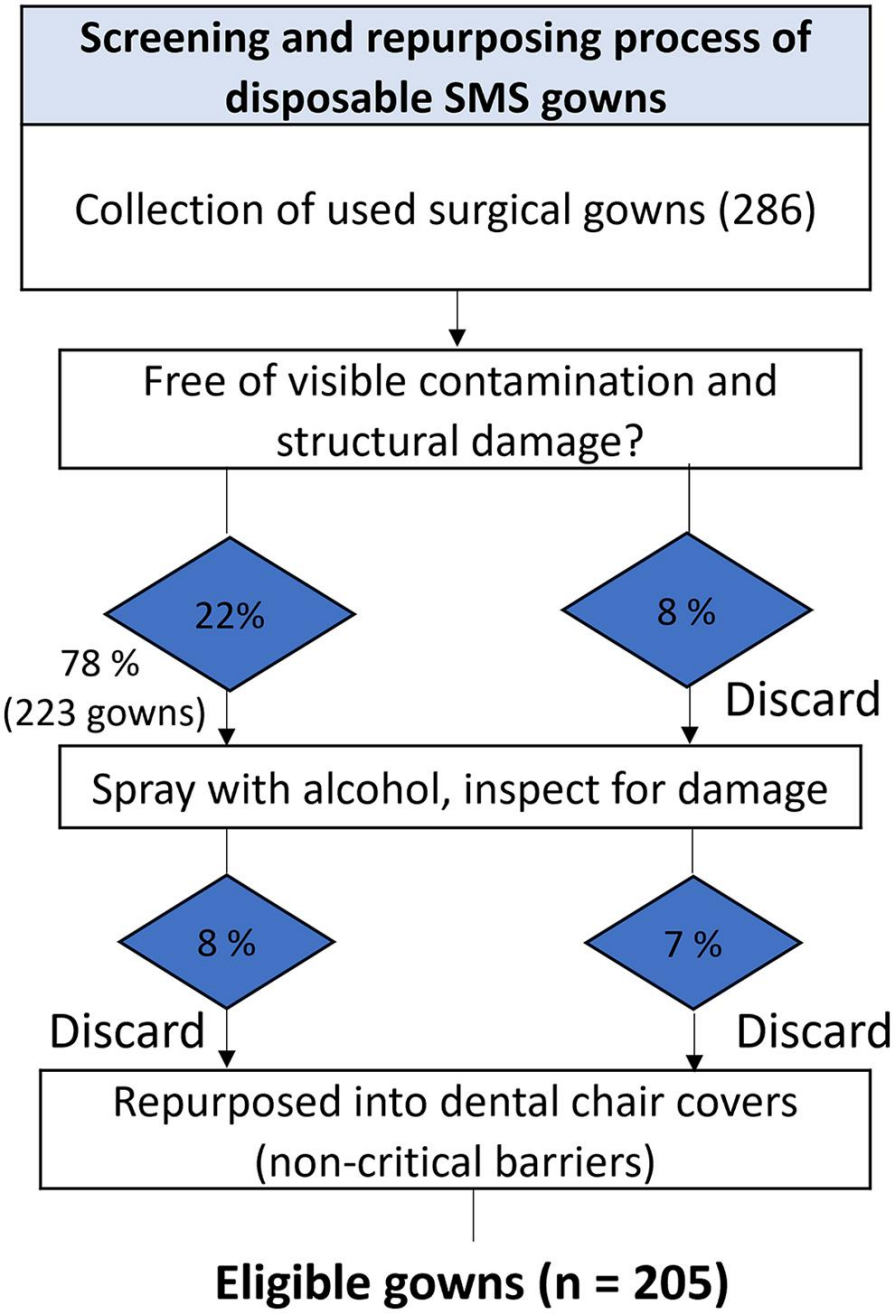
Flowchart onf the screening, decontamination, and repurposing process of disposable SMS polypropylene gowns into non-critical environmental barriers within a university dental education setting.

The overall methodological workflow of the study is summarized in Figure 1. The intervention followed a structured, multi-phase process: screening, cleaning/disinfection, and transformation. Gowns were first inspected for structural integrity. Selected items underwent a standardized cleaning/disinfection cycle: cleaning with neutral detergent, immersion in freshly prepared 0.1% sodium hypochlorite for a validated contact time, rinsing with sterile water, and air-drying in a controlled environment. To ensure the reduction of microbial load and material stabilization, items were subjected to terminal dry heat (70–75 °C). Finally, decontaminated materials were repurposed into dental chair covers, classified as non-critical surface barriers, using standardized patterns and reinforced stitching. This decontamination protocol was intended to reduce environmental contamination and stabilize material performance for non-critical applications, and was not designed to achieve sterility or enable clinical reuse of PPE.

### 2.5 Functional performance and perception assessment

The study evaluated three complementary outcome domains. Functional performance variables included material stability during clinical use, resistance to routine surface disinfection, and preservation of structural integrity following the repurposing protocol. Perception variables included participants' evaluations of environmental awareness, perceived usefulness, sustainability culture, willingness to support institutional implementation, and overall project acceptance, measured using validated 5-point Likert-scale questionnaires. In addition, an environmental outcome variable was estimated as the quantity of SMS polypropylene diverted from disposal and the corresponding preliminary reduction in production-related CO₂-equivalent emissions.

Reprocessed chair covers were assessed during routine, non–aerosol-generating dental training procedures to minimize cross-contamination risks. Assessment focused on stability, resistance to surface disinfectant wiping (e.g., alcohol or quaternary ammonium), and material integrity. User perceptions were evaluated through a validated 5-point Likert-scale questionnaire (Cronbach's alpha confirmed internal consistency) covering sustainability awareness and functional adequacy.

### 2.6 Data analysis

Quantitative data were analyzed using descriptive statistics, including frequencies, percentages, means, and standard deviations to characterize participant perceptions. To compare perception scores between students and professionals, non-parametric tests (Mann–Whitney U test) were applied, as the data derived from Likert scales are ordinal in nature. Internal consistency of the perception instrument was strictly verified using Cronbach's alpha coefficient. Qualitative responses regarding open-ended questions were analyzed through inductive thematic analysis following Braun and Clarke's framework to identify recurring patterns in sustainability barriers. Statistical analyzes were performed using IBM SPSS Statistics v26.0 (IBM Corp., Armonk, NY, USA).

### 2.7 Ethical considerations

All participants provided informed consent. The study focused exclusively on environmental sustainability, material functionality, and user perception. The study involved no patients or biological sampling and did not evaluate sterility. Repurposed materials were used exclusively as non-critical environmental barriers under routine surface disinfection protocols within an educational clinical setting.

## 3 Results

### 3.1 Participant characteristics and instrument reliability

A total of 173 participants were included, comprising 148 dental students and 25 dental professionals (Table 1). Students were predominantly female (59.5%) with a mean age of 18.9 ± 1.2 years, while professionals were mostly male (72.0%) with a mean age of 39.9 ± 8.7 years. This demographic diversity allowed for a comprehensive assessment of perceptions across different levels of clinical and academic experience. The perception questionnaire demonstrated high internal consistency, with a Cronbach's alpha of *α* = 0.82, confirming the reliability of the instrument.

**Table 1:** Sociodemographic characteristics of participants by study group.

Variable	Students (*n* = 148)	Professionals (*n* = 25)	Total (*n* = 173)
Age (years), mean ± SD	18.9 ± 1.2	39.9 ± 8.7	Not applicable
Female, *n* (%)	88 (59.5)	7 (28.0)	95 (54.9)
Male, *n* (%)	60 (40.5)	18 (72.0)	78 (45.1)
Academic/Professional level	Undergraduate students (Preclinical–Clinical)	Dental professionals	Not applicable

SD, standard deviation; n, number of participants; percentages are calculated within each study group.

### 3.2 Material flow and repurposing eligibility

From an initial collection of 286 disposable SMS polypropylene gowns, a systematic screening process was implemented. During the first visual inspection, 22% were excluded due to visible biological contamination and 8% due to structural damage. A secondary inspection involving manual integrity assessment and alcohol spraying resulted in the further exclusion of 15% of the items. Ultimately, 205 gowns (71.7% of the total collected) met all criteria for successful repurposing into dental chair covers (Figure 1).

### 3.3 Perception analysis and global acceptance

Overall acceptance was positive in both cohorts. Professionals reported significantly higher scores in satisfaction with participation (4.28 ± 0.89) compared to students (4.02 ± 0.81) (Table 2). Conversely, students exhibited slightly higher mean scores in environmental awareness (3.98 ± 0.82).

**Table 2:** Mean perception scores of the sustainability project by study group.

Domain	Students, mean ± SD	Professionals, mean ± SD
Environmental awareness	3.98 ± 0.82	3.72 ± 1.10
Perceived usefulness	3.89 ± 0.77	3.80 ± 1.08
Satisfaction with participation	4.02 ± 0.81	4.28 ± 0.89
Overall acceptance score	**3.96** **±** **0.79**	**3.96** ± **0.94**

SD, standard deviation. Scores are based on a 5-point Likert scale (1 = strongly disagree to 5 = strongly agree).

Bold values indicate the overall acceptance scores, representing the primary summary outcome of the perception assessment.

**Figure 2:**
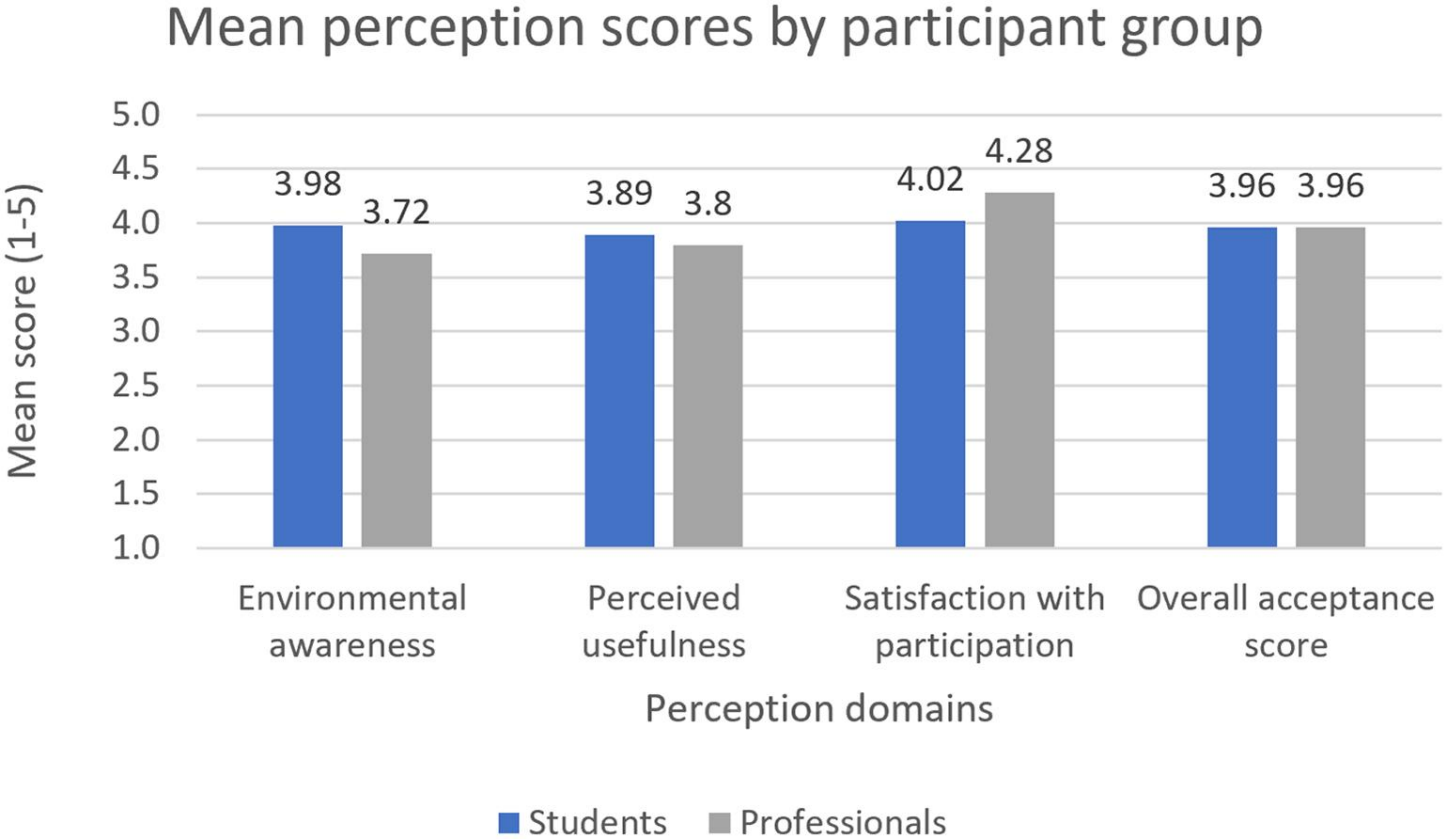
Descriptive comparison of mean perception scores (Likert scale 1–5) between dental students and professionals across sustainability-related domains in a university dental education setting.

Comparative analysis (Figure 2) indicates that professionals showed greater readiness to adopt circular textile strategies, scoring higher in indicators related sustainability culture and institutional importance. Among students, more than 60% expressed “Agreement” or “Strong Agreement” across all domains, with the highest consensus found in waste management awareness (66.8%) and willingness to participate in future initiatives (66.2%) (Table 3).

**Table 3:** Distribution of agreement levels among dental students.

Statement	Disagreement^a^, *n* (%)	Neutral, *n* (%)	Agreement^b^, *n* (%)
Importance of sustainability	10 (6.8)	42 (28.4)	96 (64.8)
Acquisition of practical skills	12 (8.1)	45 (30.4)	91 (61.5)
Waste management awareness	10 (6.8)	39 (26.4)	99 (66.8)
Product usefulness	14 (9.5)	36 (24.3)	98 (66.2)
Willingness to participate again	12 (8.1)	38 (25.7)	98 (66.2)

a Disagreement includes ’strongly disagree’ and “disagree”.

b Agreement includes “agree” and ’strongly agree’.

### 3.4 Institutional feasibility and implementation

A high level of institutional recommendation was observed, particularly among professionals (80%) compared to students (66.2%) (Table 4). The global perception index for the project among students reached 3.89 out of 5, interpreted as a “Positive” overall perception (Table 5). These results support the feasibility of scaling the initiative as a formal component of the institutional dental training program.

**Table 4:** Comparison of overall acceptance indicators between students and professionals.

Indicator	Students (*n* = 148)	Professionals (*n* = 25)
Positive perception (mean score ≥ 4), %	65.1%	80.0%
Willingness to recommend implementation	66.2%	80.0%
Overall satisfaction (mean ± SD)	3.84 ± 0.78	4.28 ± 0.89

SD, standard deviation. Positive perception was defined as a mean Likert score ≥ 4 on a 5-point scale. Percentages are calculated within each study group.

**Table 5:** Global perception index of the sustainability project among dental students (*n* = 148).

Domain	Mean score (1–5)	SD	Interpretation
Sustainability awareness	3.92	0.88	High
Practical skills acquisition	3.81	0.91	Moderate–high
Perceived usefulness of products	3.89	0.94	High
Willingness for future participation	3.93	0.90	High
Overall perception index	**3** **.** **89**	**0**.**91**	**Positive**

SD, standard deviation. Interpretation categories were defined *a priori* based on mean Likert scores (1–5): Moderate–high (3.5–3.9), High (≥4.0), and Positive indicates an overall favorable perception.

### 3.5 Estimated environmental impact

Based on the 205 gowns successfully repurposed, the environmental impact was estimated using published average weight data for disposable surgical gowns reported in a prior life cycle assessment (mean 63 g; range 41–91 g) (19). Using the representative mean weight (63 g), approximately 12.9 kg of SMS polypropylene were diverted from immediate disposal.

To estimate potential emission savings, a polypropylene production emission factor reported in international life cycle assessment studies (1,586 kg CO₂-equivalent per kg of PP) was applied (20). This value corresponds to cradle-to-gate emissions associated with virgin resin production, including feedstock purification, polymerization, and pelletization stages. Under this assumption, the diversion of these materials corresponds to an estimated reduction of approximately 20.5 kg CO₂-equivalent, with a plausible range between 13.3 and 29.6 kg CO₂-equivalent, depending on variability in gown mass.

These estimates provide a preliminary quantitative indicator of the environmental benefit associated with the intervention. However, they do not replace a comprehensive life cycle assessment that would incorporate additional factors such as nonwoven textile manufacturing processes, transportation, cleaning and disinfection stages, and end-of-life scenarios including incineration or landfill disposal.

## 4 Discussion

The present study demonstrates that a controlled repurposing strategy for disposable SMS polypropylene surgical gowns is feasible and positively accepted within a university dental setting. By utilizing standardized inspection and decontamination procedures, 71.7% of gowns originally intended for single use were successfully converted into functionally dental chair covers. These findings align with previous reports highlighting the urgent need to reduce dental solid waste and promote sustainable material management within dental practice and education (21, 22).

### 4.1 Environmental implications

The preliminary environmental estimation incorporated in this study is consistent with published life cycle assessments of disposable surgical gowns, which identify polypropylene production as a major contributor to their environmental footprint (19). By diverting material mass from immediate disposal, even at modest scales within a university dental clinic, the intervention contributes to reducing upstream production-related emissions and solid waste generation. While this estimation does not substitute for comprehensive life cycle modeling, it provides a quantitative framework supporting the environmental rationale of the intervention within an academic clinical context. These findings reinforce the relevance of operational-level circular strategies in mitigating healthcare-related material flows.

Similar concerns regarding the environmental burden of dental clinical waste have been highlighted in recent analyzes of sustainability education within dental care, where healthcare waste management has been identified as a priority domain for environmental intervention (23). These reports emphasize that educational and operational strategies must coexist in order to translate environmental awareness into measurable reductions in material flows. In this regard, the present study contributes by integrating quantitative material diversion metrics with an institutional workflow model, bridging theoretical sustainability principles with applied implementation in dental training environments.

While previous studies have examined disposable vs. reusable gown systems from a hospital-based or sterilization-centered perspective, fewer investigations have explored structured repurposing models within dental education contexts. The present study contributes by translating circular economy principles into an operational workflow tailored to university dental clinics, integrating environmental estimation, functional evaluation, and stakeholder acceptance within a single applied framework.

### 4.2 Functional viability and decontamination

From a functional perspective, the reprocessed SMS polypropylene chair covers demonstrated high structural stability, adequate resistance to displacement, and full tolerance to routine surface disinfection during non–aerosol-generating dental training procedures. A critical finding was that most material exclusions occurred during the initial visual inspection stage (22% for contamination and 8% for damage) rather than after reprocessing. This underscores the relevance of strict pre-selection criteria based on physical integrity and absence of visible contamination (24), suggesting that effective repurposing strategies for non-critical items may rely more on controlled handling protocols than on exhaustive laboratory-based testing.

### 4.3 Perception and institutional readiness

The high global perception index among students (3.89/5) and the even stronger support for institutional adoption among professionals (80%) indicate a substantial readiness for circular economy practices in healthcare environments. Professionals consistently reported higher mean scores in satisfaction and sustainability culture, suggesting that professional experience may enhance the readiness to embrace circular-economy practices (25, 26). However, given the smaller number of professionals included in the study (*n* = 25), these subgroups findings should be interpreted cautiously and considered exploratory. These results emphasize the need to transition from linear, single-use models toward circular management strategies that combine environmental responsibility with operational feasibility.

### 4.4 Educational impact and sustainability competencies

Unlike studies focused primarily on microbiological validation, this research intentionally adopted an environmentally centered and educational approach. The project functioned as an applied learning experience, fostering environmental awareness among dental students while offering a pragmatic waste-reduction strategy (27, 28). Embedding such experiences within academic clinical environments provides an opportunity to normalize environmentally responsible behaviors without compromising operational efficiency (28–30). This resonates with broader recommendations to embed sustainability as a transversal competence within healthcare education, integrating environmental responsibility into professional identity formation and clinical decision-making processes (27, 31–33). These findings are consistent with recent empirical evidence demonstrating that structured sustainability interventions within dental education significantly enhance students’ environmental awareness and willingness to engage in sustainable clinical practices (32). Furthermore, national-level surveys among oral healthcare students have reported high concern regarding dentistry's environmental footprint but limited formal curricular exposure, underscoring the need for institutionally embedded sustainability initiatives (29). Compared with these reports, the present study advances the field by coupling perception assessment with operational material repurposing and preliminary environmental quantification within a single applied framework. An educational infographic was also developed as part of the initiative to support sustainability awareness and knowledge translation among students (Supplementary Figure S1).

### 4.5 Limitations and future directions

This study presents certain limitations. First, microbiological testing was not performed because the study scope was limited to feasibility and sustainability-oriented repurposing rather than sterility assurance. Routine sterility testing may also increase resource use and laboratory waste, potentially offsetting some environmental gains. Second, the intervention was implemented within a single academic institution, which may limit generalizability. Additionally, an imbalance between student and professional participants was observed, reflecting the natural composition of the academic clinical environment. However, the relatively smaller number of professionals limits the strength of subgroup-specific conclusions. Future research should expand upon this model by incorporating life-cycle assessments and comprehensive laboratory testing to characterize the mechanical and microbiological behavior of reprocessed SMS textiles under controlled conditions. Therefore, the present findings should not be extrapolated to clinical PPE repurposing or to aerosol-generating dental procedures. Additionally, although a preliminary carbon footprint estimation was included, a comprehensive life cycle assessment incorporating energy consumption, water use, and transportation impacts is required to determine the net environmental benefit under real-world operational conditions.

## 5 Conclusions

Beyond material feasibility, the intervention demonstrated that a structured, sustainability-oriented repurposing strategy for disposable SMS polypropylene gowns can be operationalized within a university dental clinic with high stakeholder acceptance. A total of 71.7% of collected gowns were successfully repurposed into non-critical environmental barriers, indicating practical applicability under controlled inspection and handling protocols.

The inclusion of a preliminary environmental impact estimation further strengthens the sustainability rationale by quantifying material diversion and associated production-related emission reductions. While not a substitute for comprehensive life cycle assessment, this quantitative approximation provides an evidence-informed framework for institutional decision-making.

By embedding circular economy principles into routine clinical training, the initiative fostered environmental awareness and sustainability competencies among future dental professionals while maintaining operational feasibility. This integrated approach—combining functional evaluation, stakeholder perception, and environmental estimation within an academic workflow—offers a replicable institutional pilot model for dental schools seeking to reduce material waste and advance sustainability in oral healthcare.

These findings should not be extrapolated to clinical PPE reuse or aerosol-generating procedures, but rather understood as a sustainability-oriented repurposing strategy for non-critical applications within controlled educational environments.

## Data Availability

The raw data supporting the conclusions of this article will be made available by the authors, without undue reservation.

## Supplementary material

Supplementary Figure S1 Educational infographic illustrating the sustainability-oriented repurposing process of disposable SMS polypropylene gowns into non-critical environmental barriers within a university dental education context.
