# Supplementary figures and images for: Sustainable repurposing of disposable SMS polypropylene gowns in dental education: a feasibility study on material functionality and user perception
Source: Front Dent Med. 2026 Jul 20;7:1778387. doi: 10.3389/fdmed.2026.1778387 (PMC13429606; doi:10.3389/fdmed.2026.1778387)

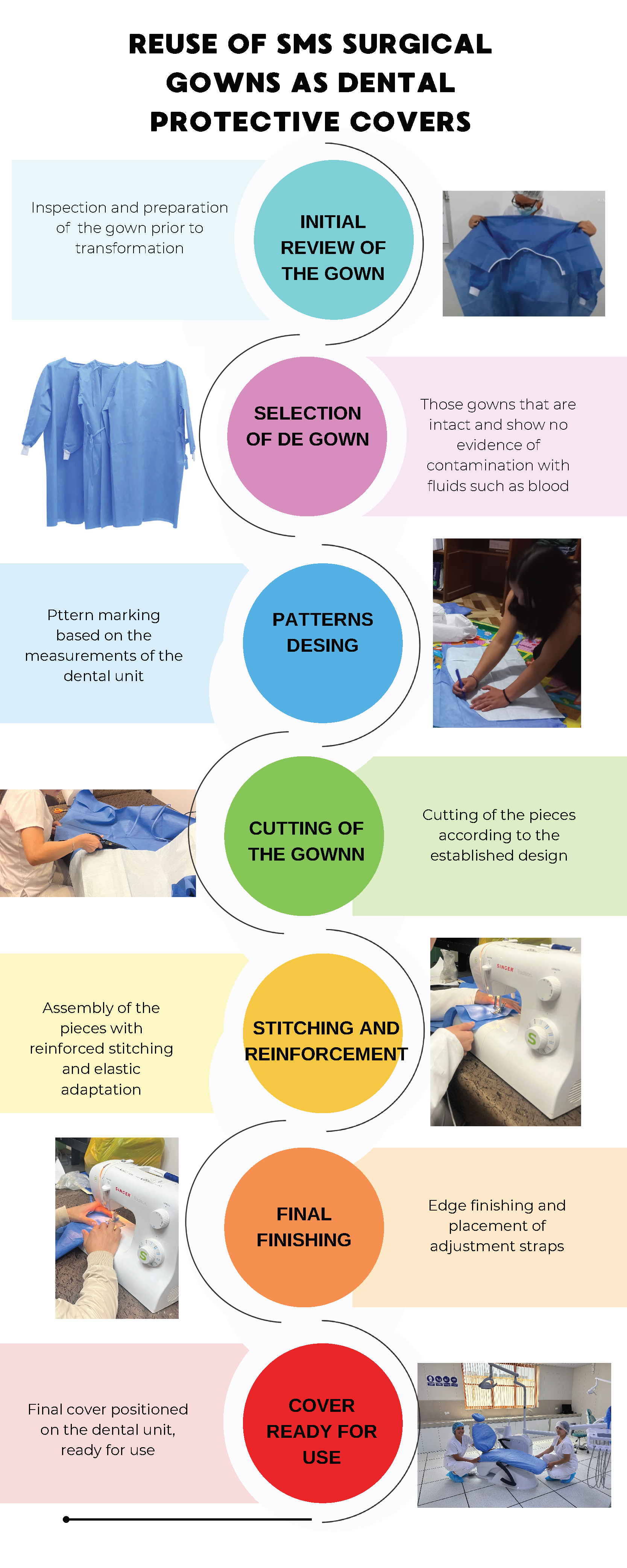

Supplement: Supplementary Figure S1 — Educational infographic illustrating the sustainability-oriented repurposing process of disposable SMS polypropylene gowns into non-critical environmental barriers within a university dental education context. [file Image1.tiff]
